# Quantum core affect. Color-emotion structure of semantic atom

**DOI:** 10.3389/fpsyg.2022.838029

**Published:** 2022-09-28

**Authors:** Ilya A. Surov

**Affiliations:** ITMO University, Saint Petersburg, Russia

**Keywords:** color, map, affect, emotion, meaning, atom, qubit, sphere

## Abstract

Psychology suffers from the absence of mathematically-formalized primitives. As a result, conceptual and quantitative studies lack an ontological basis that would situate them in the company of natural sciences. The article addresses this problem by describing a minimal psychic structure, expressed in the algebra of quantum theory. The structure is demarcated into categories of emotion and color, renowned as elementary psychological phenomena. This is achieved by means of quantum-theoretic qubit state space, isomorphic to emotion and color experiences both in meaning and math. In particular, colors are mapped to the qubit states through geometric affinity between the HSL-RGB color solids and the Bloch sphere, widely used in physics. The resulting correspondence aligns with the recent model of subjective experience, producing a unified spherical map of emotions and colors. This structure is identified as a semantic atom of natural thinking—a unit of affectively-colored personal meaning, involved in elementary acts of a binary decision. The model contributes to finding a unified ontology of both inert and living Nature, bridging previously disconnected fields of research. In particular, it enables theory-based coordination of emotion, decision, and cybernetic sciences, needed to achieve new levels of practical impact.

## 1. Introduction

Every science stands on ontological primitives, giving an ultimate answer to the question “What are we talking about?”. Elementary physics, for example, talks about material bodies, moving in space-time and interacting by contact forces. Out of such bodies, however, only inert ones (like stones on the road) follow Newtonian predictions. Others ignore these laws, doing their own business. Such misconduct is usually excused by saying that besides their material bodies, these things also have “psychology” that ruins the theory. A dedicated field of knowledge, however, is not marked by decisive success: state-of-the-art models develop diverging conceptual views, often reporting non-reproducible results (Baker, 2015; Swiatkowski and Dompnier, 2017; Oberauer and Lewandowsky, 2019). The motion of strange particles called “living” remains a mystery for science.

Modeling of cognition and behavior on quantum principles is an attempt to get out of a methodological deadlock, faced by other approaches. Surprisingly for many, this extreme move produced useful results. In particular, it allowed quantitative descriptions of cognitive fallacies and irrational behavior (Agrawal and Sharda, 2013; Ashtiani and Azgomi, 2015), regularities of natural language (Gabora et al., 2008; Melucci, 2015), non-expected cooperation and game equilibria (Alonso-Sanz, 2012; Szopa, 2021), complex collective social and economic effects (Haven, 2015; Khrennikov, 2018), and other features of human judgment, preference, and logic, challenging classical methods of modeling. Crucially, this is achieved from a unitary theoretical structure that emerges as a conceptual-theoretic framework, unifying previously disjoined areas of psychology and cognitive science (Khrennikov, 2010; Busemeyer and Bruza, 2012; Wendt, 2015; Aerts et al., 2016; Trnka and Lorencova, 2016).

This advantage of quantum theory stems from its wider ontology, as compared to the matter-energy—space-time system, mentioned above. Namely, a central element of quantum modeling—the wavefunction, usually denoted by Greek letter ψ (psi)—although coupled to both, is neither material nor energetic; as a vector in multidimensional Hilbert space, it is also beyond space and time as they are known in classical physics. The world seen through categories of quantum theory is thereby richer than possible from a mechanistic perspective (Aerts, 1999). Living behavior, invisible through classical physics, then appears as belonging to this newly added ψ-dimension of Nature, usually called “psychological.” It could be called simply “informational,” if the original meaning of this term would not be distilled from the affectively-semantic part of it (Weber, 2011; Markoš and Cvrčková, 2013); now, essentially psychological information, captured in quantum models, is better named *affective meaning* or *subjective experience* (ibid.).

The cognitive function of ψ, however, is not yet completely understood. The structure of Hilbert space in which it lives, although central to the obtained advantages, largely remains an abstract mathematical formalism. In particular, phase parameters of complex-valued elements of ψ lack psychological interpretation, necessary to get in touch with classical psychology and cognitive science. This “phase problem” also contributes to the inability of using quantum models in predictive mode, reducing their practical impact (Surov et al., 2019).

**This article** aims to establish the lacking connection by means of *color*. On the humanitarian side, color is integrated with research on emotion, perception, language, and other cognitive functions (Elliot and Maier, 2014); starting from the inception of modern psychology, it was used to explore processes of perception, sensation and feeling, memory and imagination, composition of mental categories, similarity and classification judgment, the interplay between objective and subjective aspects of human mind (James, 1890; Wundt, 1897). The semantic function of colors is found to be largely stable across cultures and epochs Adams and Osgood (1973), indicating its central position in natural thinking.

Besides this generality, color is unique among other psychological primitives by affinity to mathematical encodings. This is crucial for finding contact with quantum theory. From this side, the model of choice is the simplest quantum state—the qubit (Le Bellac, 2006, ch. 2). While maintaining key features of the quantum approach such as contextuality, superposition, and entanglement, it is unique in allowing simple geometrical representation, recently interpreted as an individual semantic space (Surov, 2021). Furthermore, the qubit structure is shown to encode elementary states of emotional experience (Surov, 2022). This link allows mapping of color to qubit states both mathematically and semantically, providing complete psychological interpretation for this particular class of quantum-cognitive states.

The generality of the obtained structure identifies the qubit as an elementary unit of affective meaning and subjective experience. Akin to the blocks of matter, central to the mechanistic worldview, this “semantic atom” is considered to play the same role in the psychological domain of Nature. This indicates a possibility for extending the “hard” physical ontology to the living part of Nature, addressing the foundational problem, noted above.

This result is approached in the following steps. First, Section 2 outlines the quantum-theoretic model of semantic space (Surov, 2022), central to the following analysis. Next, Section 3 summarizes previously established meanings of basic colors, including state of the art in color-emotion correspondence and relevant models of color. Based on that, Section 4 matches dimensions of color to that of the qubit semantic space, formalizes this matching in quantum-theoretic Hilbert-space calculus, and discusses features of the obtained map. Section 5 then establishes correspondence with the recent model of subjective experience (ibid.), describing a unified qubit-color-emotion semantic map and discussing its practical implications. Section 6 concludes the article by conceptualizing the obtained result as the core semantic unit of natural cognition.

## 2. Qubit model of individual semantic space

Following Surov (2022), the present approach considers individual cognition as serving a particular binary decision, judgment, or behavioral act faced by a subject, such as making tea or not. However trivial that may seem, in the present approach choices of this kind are the only thing one ever does in a free manner, requiring deliberate control^1^. Once a decision is made, subsequent action (in parallel with many other processes Bargh and Chartrand, 1999) unfolds automatically up to the next crossroad in behavioral algorithmics. In the tea making case, this could be, e.g., a decision whether to add sugar or not at a particular stage of the procedure. Each such occasion generates its own semantic space of subjective experience, dedicated to the resolution of the basic uncertainty.

The next subsections expand this approach in the following order. First, Section 2.1 summarizes the qubit model of subjective experience and describes the cognitive function of its basic dimensions; a detailed introduction to the mathematics in use can be found in Busemeyer and Bruza, (2012, ch. 2) and Haven and Khrennikov, (2013, ch. 4). Next, Section 2.2 detalizes the process-semantic structure of the qubit's phase dimension, central for the following analysis. Based on that, Section 2.3 shows how these dimensions accommodate basic emotion classes. Finally, Section 2.4 generalizes this model to describe the mixing of emotional experiences, necessary to account for the mixed colors afterward.

### 2.1. Representation of contexts by qubit states

Consider an individual bound to make a choice between two mutually exclusive cognitive-behavioral alternatives such as DO or NOT DO (some action, e.g., to *take a walk*), TRUE or FALSE (estimation-judgment e.g., of statement *weather is good*), and YES or NO (decision-answer e.g., to question *are you healthy?*). In the cognition of a subject, all information used to make this decision, called *context*, is represented by a *qubit* state (Jaeger, 2007, ch. 1.2).


(1) $$|\psi \rangle =c_{0}|0\rangle +e^{i\phi }c_{1}|1\rangle =\cos\frac{\theta }{2}|0\rangle +e^{i\phi }\sin\frac{\theta }{2}|1\rangle ,$$

where 0 and 1 label decision alternatives, *c*_*i*_ are real-valued coefficients, and 0 ≤ ϕ < 2π is the phase parameter. In Dirac notation (Kasirajan, 2021), angle brackets |·〉 denote column vectors, so that qubit state |ψ〉 is a vector in two-dimensional (Hilbert) space, formed by basis vectors


(2) $$|0\rangle =\left[\begin{array}{c} 1\\ 0 \end{array}\right],\;|1\rangle =\left[\begin{array}{c} 0\\ 1 \end{array}\right],\;|\psi \rangle =\left[\begin{array}{c} c_{0}\\ e^{i\phi }c_{1} \end{array}\right].$$

**Figure 1:**
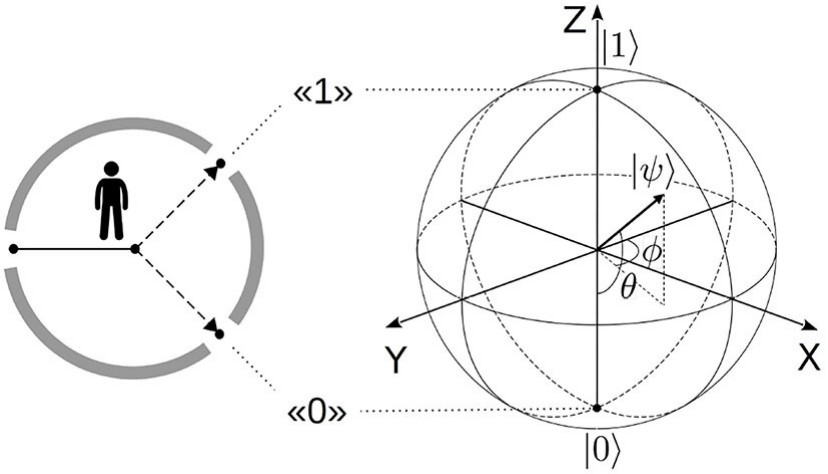
Qubit model of context representation (Surov, 2021). Individual facing a two-way alternative represents all available information (context), shown by gray bars, relative to this decision by qubit state |ψ〉 (Equation 1). All such states belong to a Poincaré (Bloch) sphere, built on the poles corresponding to basis alternatives |0〉, |1〉, as shown on the right. The sphere functions as cognitive-semantic space, subjectively constructed specifically for this decision task.

With coefficients *c*_*i*_ parameterized by (polar) angle 0 ≤ θ ≤ π, state (Equation 1) is visualized by a unit vector, pointing from the origin of three-dimensional space to the surface of a unit-radius (Poincaré-Bloch) sphere as shown in Figure 1. Unlike standard Euclidean geometry, orthogonality of the qubit states corresponds to the opposite orientation, as seen for basis vectors 〈0|1〉 = 0, pointing to the poles of the sphere.

By representing context in relation to the basis decision to be made, the qubit state (Equation 1) encodes its *meaning* in subjective experience of the considered person (Surov, 2022).

#### Polar angle: Evaluation

Probabilities, with which an individual chooses basis alternatives from the experiential state (Equation 1), are defined by standard quantum-theoretic Born's rule


(3) $$\begin{array}{l} p_{i}=|\langle \psi |i\rangle {|}^{2}=c_{i}^{2},\;p_{0}+p_{1}=\cos^{2}\frac{\theta }{2}+\sin^{2}\frac{\theta }{2}=1, \end{array}$$

and measured statistically for an ensemble of identically (indistinguishably) staged experiments. In the above expression, 〈ψ|*i*〉 denotes the inner product of basis vectors |0〉, |1〉 with complex-conjugate (Hermitian) transpose of state vector (Equation 2)


(4) $$\begin{array}{l} {\langle \psi |=|\psi \rangle }^{†}=\left[\begin{array}{cc} c_{0} & e^{-i\phi }c_{1} \end{array}\right]. \end{array}$$

**Figure 2:**
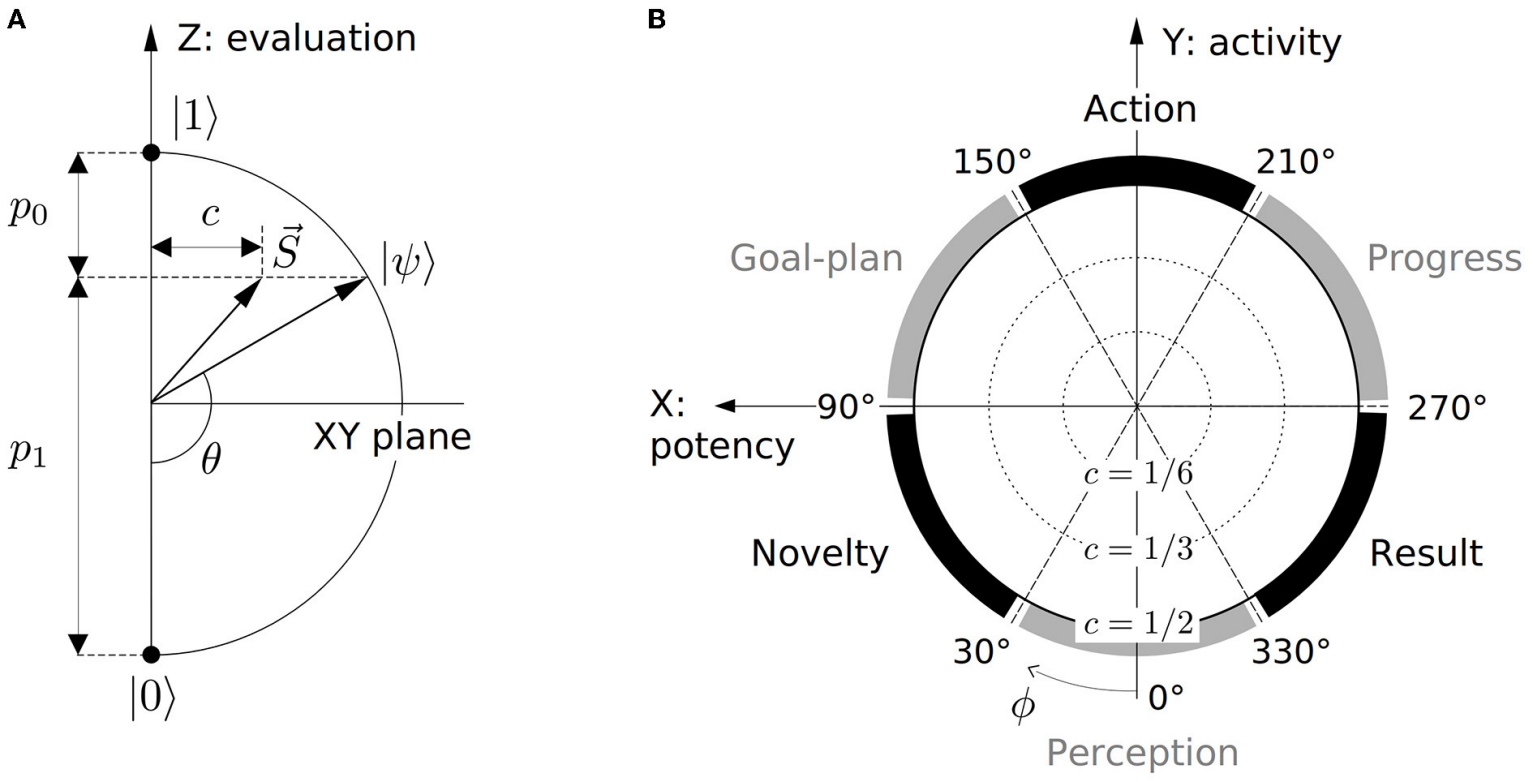
Dimensions of the qubit space of subjective experience. **(A)** Vertical section of the Bloch sphere with Z-axis encoding subjective evaluation of contexts. Z-component of both pure (Equation 1) and mixed (Equation 8) state vectors defines observable probabilities *p*_*i*_. Coherence *c* quantifies control of a subject over the resolution of the basis decision alternative. **(B)** Horizontal section of the Bloch sphere. Process-semantic classes of contexts Perception, Novelty, Goal-plan, Action, Progress, and Result demarcate the qubit's azimuthal dimension ϕ. Pure and mixed states of each class occupy the perimeter and interior of the corresponding azimuthal sector.

Decision probabilities 0 ≤ *p*_*i*_ ≤ 1 quantify conduciveness of the represented context for the basis decision alternatives as considered by a subject. Angle θ thus functions as the *evaluative* dimension of the qubit state (Equation 1), shown in Figure 2A. The top and bottom Bloch hemispheres in Figure 1 then accommodate representations of positively and negatively evaluated contexts, respectively.

#### Example

Consider a decision whether to have a cup of tea (1) or not (0). It generates a task-specific Hilbert space of individual experience of a decision maker, accommodating subjective representations of all possible decision contexts. In this space, the context described, e.g., by a single word *hurry* is likely to be mapped to the qubit state (Equation 1)


(5) $$|\text{hurry}\rangle =\cos\frac{\theta }{2}|\text{no tea}\rangle +e^{i\phi }\sin\frac{\theta }{2}|\text{tea}\rangle .$$

Since hurry is not conducive for tea parties, polar angle θ must be less than 90°, so that $p_{\text{no tea}}=\cos^{2}\left(\theta /2\right)>\sin^{2}\left(\theta /2\right)=p_{\text{tea}}$.

As a typical result of having tea, the context *relaxation*, analogously, is likely to be mapped to the upper Bloch hemisphere with θ > 90°. The context *dog*, in contrast, has no conventional relation with tea. If an individual had no personal reason to establish one, the representation will fall near the equator of the sphere (or near its equatorial section, as further discussed in Section 2.4).

### 2.2. Azimuthal phase: Process order

According to Equation (3), the azimuthal phase of qubit 0 ≤ ϕ < 2π is not related to decision probabilities and subjective favorability of contexts they encode. In contrast, it allows the organization of multiple contexts in a subjectively meaningful order. This function is uniquely supported by the circular topology of ϕ, isomorphic to cyclical processes such as a year and day-night cycles, shaping the activity of living organisms in natural environments (Surov, 2022). This archetypal structure is also reflected by models of cognitive and socio-affective development (Young, 2022), cybernetic control loops (Sanz et al., 2012), and life cycles of complex systems (Hurst and Zimmerman, 1994; Dufour et al., 2018).

#### Process-semantic structure

In line with the overall discreteness of human cognition (Zipf, 1945; Rosch, 1973; Tee and Taylor, 2020), the process dimension is divided into distinct stages, facilitating recognition and categorization tasks. The number of stages is a matter of convenience, varying for different applications. Following (Surov, 2022), this article uses three primary and three secondary stages including **perception**, **novelty**, **goal-plan**, **action**, **progress**, and **result**. The resulting six-part structure, dividing the azimuthal angle ϕ into equal ranges of 60° each, is shown in Figure 2B.

After discretization, the qubit's azimuthal dimension functions as a process-semantic template for subjective representation of contexts. Since favorability is defined only for specific content, this structure itself is evaluatively-neutral. Allocation of the contexts in this template then constitutes a causal model, imposed by a subject on them relative to the basis decision alternative (Cheng, 1997; Hubbard, 2012).

#### Example continued

Continuing the tea-making case, the contexts are now allocated to different process stages in addition to their favorability considered above. In Equation (5) these process-semantic classes specify the phase angle ϕ according to the scheme shown in Figure 2B. Namely,

1. Contexts of the **perception** class include conditions and observations, motivating consideration of the basis decision alternative. In the tea-making decision, this could be, e.g., the subjective reflection of one's psycho-physiological state.
2. Contexts of the **novelty** class describe and analyze new factors, revealed during perception, like tiredness, fatigue, or thirst after intensive work.
3. Subjective intention to eliminate this pressing factor by having a drink constitutes the **goal-plan** stage. This includes a plan of where to get the necessary materials and tools, and how to use them to achieve the goal.
4. Implementation of this plan maps to the **action** stage. This includes efforts on getting a teapot, cups, and tea brewing, boiling the water, serving the table, etc.
5. Contexts of the **progress** class describe intermediate results and adjustment of the action according to the received feedback. This can be, e.g., taste and flavor of the drink and their subjective estimations.
6. As the **result** stage, the achieved invigoration and relaxation (or absence of them) might conclude the process cycle. This class of contexts also accommodates delayed consequences and the aftermath of the action, possibly sharing them with the **perception** stage of the following process cycles.

Besides this choice and mapping of contexts, of course, many others are possible. The same tea, for example, might be used to facilitate a conversation. Thirst and flavor then may be of minor significance and the same process stages would be populated by different contexts. In each case, the whole cycle constitutes a causal model of selected contexts, constructed by the subject of the basis decision alternative.

#### Algorithms for context mapping

As seen from the above examples, cognitive algorithms used to construct qubit states can be quite complex. For an other basis, e.g., to take a medicine (1) or not (0), mapping of similar contexts would require very different knowledge. The diversity of human behavior and complexity of the corresponding cognition thereby makes context mapping for each decision basis largely unique.

In elementary quantum-physical systems, in contrast, the context-mapping algorithms are the same for all practiced decisions, often allowing compact analytical expression (Feynman et al., 1964, ch. 5, 6). Accordingly, knowing a qubit state of a “spin-1/2” particle in any given basis allows physicists to find its state for any other basis by corresponding rotation of the Bloch sphere. Whether some analogous procedure is possible for macroscopic individuals is an open question.

### 2.3. Emotions as classes of qubit states

In living organisms, innate system of behavioral control is based on affectively-semantic states (Peil, 2014; Lemke, 2015; Salvatore et al., 2022), which in the case of humans are experienced as *joy, thrill, rage, zeal, bliss, fear*, etc. Identification of behavior with decision-making practice^1^ aligns affective semiosis with quantum-theoretic formalism (Surov, 2022). Human emotion, in particular, appears as a special case of subjective context representation, formalized by the qubit state math outlined above. This section introduces features of this model, necessary for the following analysis.

#### Process-value classes of emotion

According to Section 2.1, the model considers emotional experiences as defined by the evaluative dimension θ and the process-semantic dimension ϕ. Major emotions (Tomkins and Mccarter, 1964; Izard, 1977; Lazarus, 1991; Ekman and Davidson, 1994)

*happiness-joy, anger, sadness-grief*, *love, fear, hate, excitement, stress, depression, frustration, disgust, embarrassment-guilt, anxiety-worry, jealousy-envy, calmness, boredom, interest-surprise*,

then label classes of qubit states, defined by ranges of these two dimensions.

In particular, *joy* and *sadness* are most appropriate at the Result stage and not at Novelty or Action, when there is nothing yet to estimate; *acceptance* and *disgust* are experienced at the Progress stage as a feedback to the previous Action. The Action itself, in contrast, is facilitated by highly energetic *aspiration, passion, zeal*, and *rage*-type emotions, inappropriate for estimation of the Result or recognition of a Novelty. Instead, the latter is accompanied by *curiosity, interest, confusion, startle*, or *fear*. Each process stage thereby defines a class of emotional experiences, including both positive and negative ones.

In each process-semantic class, positive and negative emotions are discriminated by the polar angle θ, occupying the top (θ > 90°) and bottom (θ < 90°) halves of the Bloch sphere, respectively. For example, *anxiety, startle, fear*, and *horror* are increasingly negative experiences of Novelty, differentiated by polar angle decreasing from near the equator θ = 90° toward the south pole θ = 0°. For presentation, all such negative experiences of Novelty-class contexts with ϕ ≈ 60° are assembled into a single emotional prototype of *fear*.

#### Qubit-emotion map

**Figure 3:**
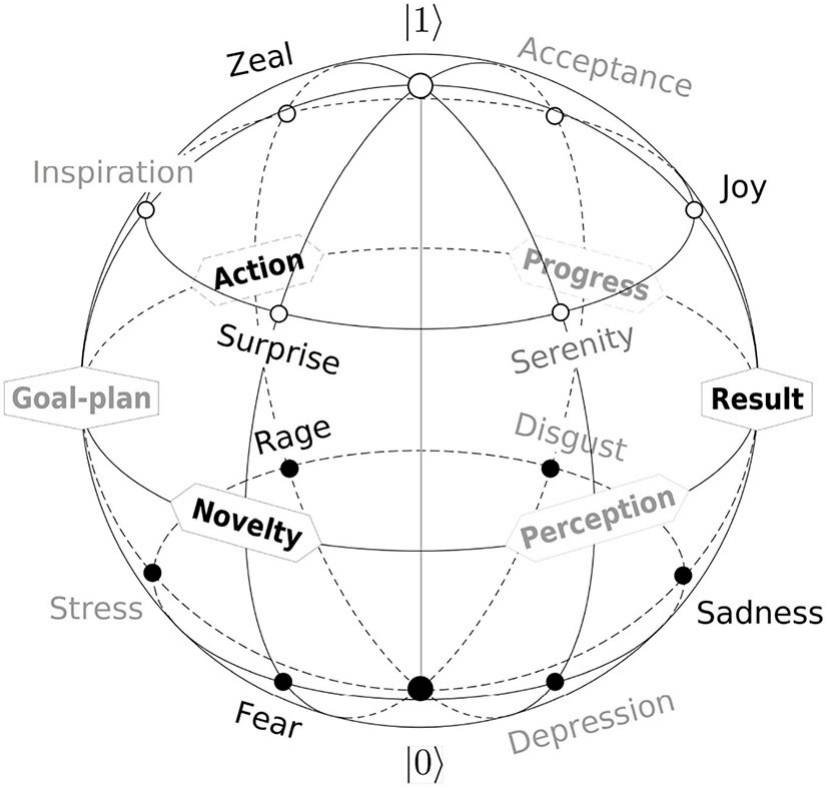
The qubit model of subjective experience in the Bloch-sphere representation. Each of three primary (black) and three secondary (gray) process stages, shown in Figure 2B, generates one positive and one negative emotional prototype. Positive and negative experiences occupy the northern (θ > 90°) and southern (θ < 90°) Bloch hemispheres according to Figure 2A. Modified from Surov (2022).

The resulting map of prototype emotions to the Bloch sphere is shown in Figure 3. The primary process Novelty, Action, and Result generate six major emotional classes *fear*-*surprise, anger*-*zeal*, and *sadness*-*joy*, previously identified by Shaver et al. (1987). Secondary stages, shown in gray, are less expressive so that the corresponding emotional terms are lower in typicality rating (Fehr and Russell, 1984).

The spherical geometry of the qubit model generalizes well-known circular models of emotion. The most popular valence-arousal circumplex (Russell, 1980; Barrett and Russell, 1998), in particular, is a section of the Bloch sphere by the Y-Z plane. These and similar models (Hevner, 1936; Schlosberg, 1952; Scherer et al., 2013), thus, appear as empirically discovered marginals of the qubit model of subjective experience (Surov, 2022).

### 2.4. Mixed states

The above theory accounts for an ideal situation when basis behavioral alternatives are well defined, the subject is in full control over their choice, and is able to include the perceived context in a single cognitive representation. In realistic cases, several pure states (Equation 1) are blended with probability-weights *P*_*i*_ in a *mixed* qubit state


(6) $$\hat{\rho }=\sum_{i}P_{i}|\psi_{i}\rangle \langle \psi_{i}|,\;\sum_{i}P_{i}=1$$

representing a “free-floating” experience directed at some context (object) or nowhere at all, as typically considered in emotion science (Russell and Barrett, 1999; Fontaine et al., 2013; Barrett et al., 2016).

According to the function of outer products |ψ_*i*_〉〈ψ_*i*_|, Equation (6) is a two-by-two matrix. It is always representable in the form


(7) $$\begin{array}{l} \hat{\rho }=\left[\begin{array}{cc} p_{0} & c\cdot e^{-i\phi }\\ c\cdot e^{i\phi } & p_{1} \end{array}\right]=\frac{1}{2}\left[\begin{array}{cc} 1-z & x-iy\\ x+iy & 1+z \end{array}\right], \end{array}$$

where *p*_0_, *p*_1_ are decision probabilities, *c* is real-valued parameter considered below, and *i* is imaginary unit as in Equation (1). In the latter part of Equation (7), *x, y, z* are real-valued components of a three-dimensional (Stokes) vector (Jaeger, 2007, ch. 1.3)


(8) $$\begin{array}{l} \vec{S}=\left[\begin{array}{c} x\\ y\\ z \end{array}\right],\;|\vec{S}{|}^{2}=x^{2}+y^{2}+z^{2}\leq 1 \end{array}$$

representing mixed subjective experience, encoded by qubit state (Equation 6).

Mixed states (Equations 6, 7) generalize pure states (Equation 1) for which inequality in Equation (8) is saturated. Geometrically, this corresponds to using state vectors $\vec{S}$, occupying the interior of the Bloch sphere along with its surface. In particular, suppression of non-diagonal element *c* of the matrix (Equation 7) below pure-case limit $\sqrt{p_{0}p_{1}}$ partially projects the state vector to the Z axis as sketched in Figure 2A.

#### Coherence: Freedom to affect

Parameter *c* in Equation (7) is *coherence* of the qubit state (Baumgratz et al., 2014). It measures an ability of an individual to *affect* the resolution of the basis decision alternative, which may be called subjectness or subjective freedom. Full freedom corresponds to pure states (Equation 1) with maximal value *c* = 1/2, achieved for neutrally-evaluated experience with *p*_0_ = *p*_1_ = 1/2. In this case, contexts are mapped to the equator of the Bloch sphere, allowing for maximal resolution of the process-semantic stages.

Zero freedom *c* = 0 means that this alternative is either already resolved, or that the considered subject is unable to (meaningfully) affect its resolution (for example, due to the lack of appropriate process-semantic model, structuring the contexts). Accordingly, subjective representation of the contexts by a pure state is either useless or impossible due to limitations of an individual. In both cases, representation space reduces to the diameter of the Bloch sphere, visualizing classical binary uncertainty (Aerts, 1999; Surov, 2021).

Intermediate regimes of limited coherence are modeled by squeezing the Bloch sphere by factor *c* in X and Y directions. The resulting cognitive-semantic spaces are visualized by ellipsoids of rotation, built on the same poles |0〉 and |1〉.

#### Collapse

The uncertainty is resolved by a decision act, in which one of the previously superposed potential states comes to being, while the other is irreversibly discarded. Maintenance of the qubit representation is of no more use; the experiential space collapses with all involved contexts ceasing to make sense for the considered individual and basis. This process, known in physics as collapse of the wavefunction (Jaeger, 2017), is modeled by sending the coherence of the qubit state (Equation 7) to zero, as shown in Figure 2A. The result is a fully mixed state


(9) $${\hat{\rho }}_{\text{class}}=p_{0}|0\rangle \langle 0|+p_{1}|1\rangle \langle 1|,$$

describing ignorance of an already existing experimental outcome equivalently to the classical two-event probability space (Kolmogorov, 1956).

For the example of Sections 2.1 and 2.2, mixture (Equation 9) describes probabilistic guess (i) of a person ignorant of whether the tea party happened or not in the past, and also (ii) of a person with no control over the resolution of the same uncertainty (Equation 5) in the future.

#### Cartesian axes

By occupying the interior of the Bloch ball along with its surface, mixed states of subjective experience are encoded by three parameters, adding coherence *c* to spherical angles θ and ϕ. Equivalently, mixed states are defined by components *x*, *y*, and *z* of Stokes vector (Equation 8). These coordinates correspond to orthogonal Cartesian axes, shown in Figure 1, which also have definite cognitive functions.

Z component of Stokes vector (Equation 8) is uniquely defined by decision probabilities


(10) $$\begin{array}{l} -1\leq z=p_{1}-p_{0}\leq 1. \end{array}$$

Geometrically, point *z* is the projection of pure (Equation 1) and mixed (Equation 8) state vectors to the diameter of the Bloch sphere, with the resulting segment's lengths defining probabilities *p*_0_ and *p*_1_ according to Figure 2A. In particular, probability *p*_1_ = (*z* + 1)/2 is minimal for contexts with *z* < 0, favoring outcome |0〉, and maximal for contexts with *z* > 0, favoring |1〉. Accordingly, the Z axis generalizes the *evaluative* function of polar angle θ to both pure and mixed experiences.

Cognitive function of the X and Y axes is defined by their position in the process cycle, shown in Figure 2B. Y-axis quantifies subjective *activity* of contexts, which is maximal at Action and minimal at Perception process stage. X-axis quantifies subjective freedom, openness, or *potency* of the context, defining how strongly its variation would influence subsequent process stages. It is maximal for contexts describing subjective Goals and minimal just before seeing the Result when the process is maximally predetermined.

Z, X, and Y axes are thus identical to evaluation, potency, and activity factors of classical semantics (Osgood, 1962; Osgood et al., 1975; Surov, 2022).

## 3. Semantics of color

This section outlines state of the art in studies of color semantics, used in the following analysis. Section 3.1 summarizes qualitatively established meanings of main colors. Sections 3.2 and 3.3 then introduce basic color models, including Hering's and Young-Helmholtz's, leading to formalized spaces of color semantics. Finally, Section 3.4 considers these results in relation to the purpose of this article and identifies their principal drawback, requiring a more accurate account, presented in the next section.

### 3.1. Association studies

Semantic function of colors was recognized from the beginning of psychology. Qualitative studies indicated, that different colors are consistently associated with particular qualities and psycho-physiological states (Goldstein, 1942; Odbert et al., 1942; Aaronson, 1971; Adams and Osgood, 1973; Luscher, 1979; Frumkina, 1984; Kaiser, 1984; Wierzbicka, 1990; Hemphill, 1996; Soldat et al., 1997; Petrenko and Kucherenko, 1998; Zentner, 2001; Hill and Barton, 2005; Steinvall, 2007; Clarke and Costall, 2008; Kudrina, 2011; Niazi et al., 2015; Gilbert et al., 2016; Sutton and Altarriba, 2016; Yanshin, 2017; Fugate and Franco, 2019; Lyashchuk et al., 2021; Goethe, 1840, part VI), largely holding across languages and cultures (Osgood, 1960; Osgood et al., 1975; MacLaury et al., 2007; Heise, 2010; Jackson et al., 2019). A stable part of such associations for main colors, abstracted from these studies, is summarized in Table 1. **Red**, for example, is warm, active, strong, expanding, attractive, and dangerous. **Blue**, in contrast, is cool, passive, contracting, withdrawn, and free. **Green** is stable, sustaining, defensive, and peaceful. **Yellow** is shiny, happy, optimistic, and light. **White** is light, good, pure, and high. **Black** is bad, dark, deadly, and down.

**Table 1:** Associative meaning of main colors.

**Color**	**Associates**
Red	Fire, blood, power, action, heat, expansion, energy, force, attraction, Eros, danger, fight, aggression, stress
Green	Vegetation, harmony, balance, stability, Nature, patience, peace, equanimity, rest, respect, satisfaction, defense
Blue	Water, coolness, calmness, passivity, freedom, clarity, Logos, wisdom, intelligence, discretion, separation, alienation, distance
Violet	Mystery, magic, transformation, ceremony, luxury, richness, royalty, majesty, dignity, individuality, will
Yellow	Sun, shine, glow, radiance, happiness, kindness, divinity, lightness, optimism, openness
White	Light, clarity, purity, goodness, divinity, sincerity, emptiness, consciousness, future, life
Gray	Indifference, ignorance, non-involvement, neutrality, mediocrity, closeness, weakness, inertia, lethargy
Black	Evil, negation, protest, badness, darkness, chaos, night, unknown, destruction, dirt, oppression, death

#### Natural prototypes

Cross-cultural associative stability of colors ascends to the basic environmental factors and the practical significance they represent. Red (R), yellow (Y), green (G), and blue (B), in particular, are abstracted from natural prototypes of fire-blood, the Sun, vegetation, and sky-water, respectively (Wundt, 1897; Wierzbicka, 1990). These colors, however, are secondary with respect to (macro) white and (macro) black, forming a primary distinction pair (Wierzbicka, 1990). Due to the stability and psychologically-experiential nature of most associations, color is identified as an integral part of an archetypal system of affective meaning, underlying cognition and behavior of humans (Wierzbicka, 1990; Yanshin, 1996, 2017; Borisova, 1997; Serov, 2004; Bazyma, 2005).

### 3.2. Basic color theories

Regularities of color experience and function were represented geometrically since antiquity (Kuehni, 2003; Shamey and Kuehni, 2020). Major variables of color are commonly identified as *lightness* (brightness, luminosity) and *hue* (chromaticity, tone). The former has extremes in obvious prototypes of *black* and *white*, mixtures of which define a one-dimensional continuum of achromatic colors. Chromaticity, in contrast, is less agreed upon. While commonly accepted to have circular organization dating back at least to Newton, the numbers and allocations of basic colors differ.

According to the Young-Helmholtz theory, any color is composed of Red, Green, and Blue, detected by specialized color-sensitive cells in the retina (Wooten and Miller, 1997). This RGB triple, sometimes translated to the complementary Cyan, Magenta, and Yellow, underlies printing and image-processing technologies (Fortner and Meyer, 1997; part II). An alternative triad of basic colors exchanges green for yellow, resulting in the color circle based on Red, Yellow, and Blue (Itten, 1974).

The difficulty to see yellow as a combination of red and green motivated E. Hering to add it to the basic RGB (Hering, 1920). The resulting model defines color based on the two color pairs, blue-yellow and green-red, perceived as opposite^2^. These oppositions are commonly visualized as two orthogonal axes, forming the basis of the chromatic circle. Together with white and black, this defines color by three orthogonal dimensions (Hård and Sivik, 1981; Lindsey et al., 2020).

This scheme, however, was also found imperfect. In perceptually uniform color space, in particular, red-green and yellow-blue were found to deviate from opposite positions in the circle (Jameson and D'Andrade, 1997). A. Muncell addressed this problem by adding purple to form his set of five chromatic primaries (Munsell, 1912).

### 3.3. Formalized color spaces

Color space is quantified by the method of semantic differential, in which color patches are estimated by a subject group in a set of bipolar scales (e.g., good-bad, hot-cold, weak-strong, light-heavy, bright-dark, near-far, soft-hard, tensed-relaxed, static-dynamic, beautiful-ugly, deep-shallow, full-empty, stable-unstable, etc.) (Osgood, 1952). It is estimated that 70–90% of judgment statistics are explained by factors such as *lightness, tension*, and *temperature*, forming classical semantic space in the color domain (Osgood, 1960; Oyama et al., 1962; Williams et al., 1970; Adams and Osgood, 1973; Ou et al., 2004; Gao et al., 2007; Solli and Lenz, 2011; Sutton and Altarriba, 2016). One-to-one alignment of these factors to the classical dimensions of affective meaning *evaluation, potency*, and *activity* confirms semantic function of colors, seen from Table 1.

#### HSL color solid

**Figure 4:**
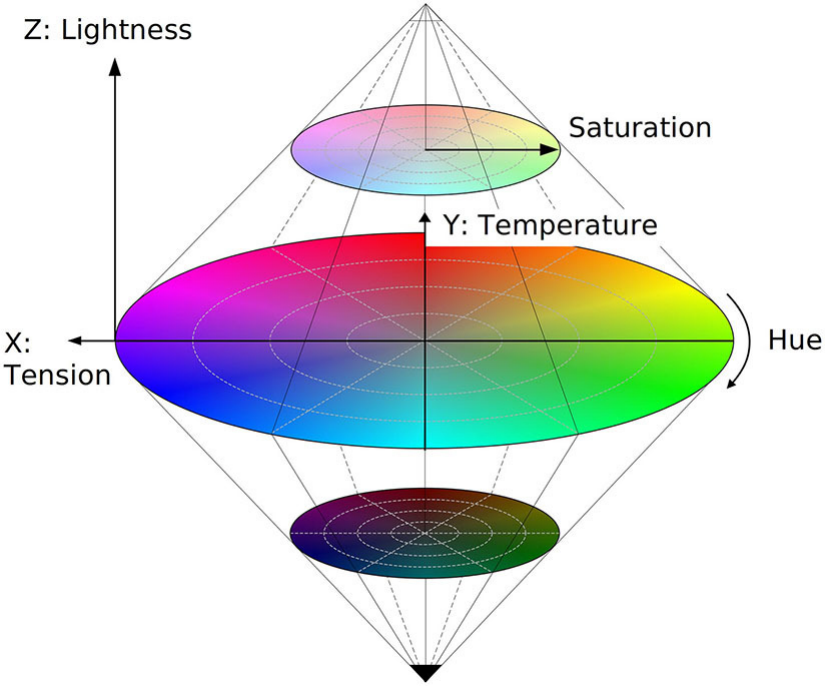
Hue-saturation-lightness (HSL) color solid. *Lightness* defines the projection of color to the black-white axis, while hue and saturation are polar coordinates of the equatorial plane, equivalently defined by Cartesian axes *temperature* and *tension*.

More intuitively, three-dimensional color space is formalized in the hue—saturation—lightness (HSL) model (Guilford, 1940; Levkowitz and Herman, 1993; Tian-Yuan, 1995; Ibraheem et al., 2012). Hue and saturation are polar coordinates in the *tension*—*temperature* plane as shown in Figure 4. Saturation is the radial distance from the achromatic lightness (Z) axis, quantifying the proximity of color experience to a gray of the same lightness. Hue is an angular dimension, the circularity of which is also established experimentally (Odbert et al., 1942; Helm, 1964; Bonnardel and Pitchford, 2006), confirming the intuition behind chromatic color circles. Hue, saturation, and lightness thus are cylindrical coordinates of the Cartesian system just mentioned, with white and black bases of the cylinder collapsed to single polar points as shown in Figure 4.

In its basic idea, the HSL model does not restrict the number and location of the main chromatic colors. Whether three, four, or Newton's seven, all of them find a place at the equator of the color solid, discretizing it accordingly. The bi-cone topology of the HSL model shown in Figure 4 is, therefore, commonly considered a proper representation of color experience in human cognition (Churchland, 2007).

With six basic colors, the HSL model is recently used to develop its quantum-inspired version, QHSL (Yan et al., 2021c). By using the qubit as a representational structure, the model connects color to the methods of quantum information, applying them to the image processing tasks (Yan et al., 2021c). QHSL, however, focuses on the formal side of the color representation without considering its semantic function, central to the present study. In this respect, the models are further compared in Section 4.4.2.

### 3.4. Naive alignment with the qubit semantics

The structure of the HSL color solid suggests obvious alignment with the qubit semantic space (Section 2). Namely, white and black map to the positive |1〉 and negative |0〉 poles of the Bloch sphere, Figure 1, such that lightness aligns with the evaluative Z dimension of the qubit. Accordingly, hue and saturation correspond to the azimuthal phase ϕ and coherence *c* of the mixed state, Equation (7). Desaturated colors inside the HSL solid (Figure 4) intuitively map to the interior of the Bloch sphere. Pure chromatic colors then appear at the equator of the Bloch sphere, possibly demarcating it to four categories red, yellow, green, and blue (Section 3.2). Opposing R-G and Y-B pairs then could match to the X and Y axes of the Bloch sphere.

#### Perceptual inaccuracy of HSL color model

This correspondence, however, has a drawback that complicates building a quantitative model of color semantics on this basis. By placing yellow on the same horizon as red, green, and blue, this model ascribes it to the same level of evaluation and lightness. The falsity of this is clearly experienced after several minutes of work with colored text. In fact, yellow (RGB=110) makes a visibly weaker contrast with white (111), as compared to red (100), green (010), and blue (001). Quantitatively, the perceptual lightness of yellow amounts to 8 out of 9, whereas red, green, and blue range from 4 to 5 (Berlin and Kay, 1975, p.8); similar results are reported in Boynton and Olson (1987), Hardin (1988), Izmailov and Sokolov (1991).

The reason for this difference is revealed by inspection of the natural prototypes and associations of colors summarized in Section 3.1. In contrast to red, green, and blue, associations of yellow (Table 1) are almost entirely positive as befits its natural prototype, the Sun. The same positivity is typical for white, to which yellow is also close semantically: things called white like bread, milk, and skin are yellowish in fact. The color temperature of the Sun (near 6,000°), accordingly, is that of white light, seemingly dropping to yellow only near sunrises and sunsets.

The same difference in lightness is readily observed for cyan (RGB=011) and magenta (101). In the standard HSL model of color shown in Figure 4, therefore, hue is orthogonal to lightness formally but not experientially. While acceptable for color-coding, introduction of color meanings as made above, and illustrative use, a reliable representation of color semantics needs a more accurate basis developed below.

## 4. The qubit-color map

This section develops the requested mapping of colors to qubit state space as follows. First, Section 4.1 explains the choice of the basic colors. Section 4.2 algebraically maps them to the qubit states, demonstrating the underlying geometrical principle. Section 4.3 then extends this principle to arbitrary colors and qubit states. Finally, Section 4.4 discusses the features of the developed map.

### 4.1. The basis color set

The minimal number of contexts, requiring the azimuthal phase ϕ for their qubit representation (Equation 1), is three. Such triple then constitutes a minimal carrier of subjective meaning as defined in Section 2 (Surov, 2021). This fundamental feature of quantum theory thereby suggests building the qubit model of color on three basis states.

Among two popular triples RGB and RYB (Section 3.2), the latter carries undesired asymmetry due to the outstanding lightness of yellow, discussed in Section 3.4. For this reason, the present model builds on the RGB color triad. In fact, only this choice aligns with semantics of the qubit space (Section 2.2), allowing one-to-one mapping to the primary process stages shown in Figure 2B. Namely,

- Red: Action
  The most energetic color obviously corresponds to the most active process stage. Force, expansion, power, passion, aggression, and fight are unambiguous associates of action, but not of Novelty or Result.
- Green: Result
  Equilibrium, peace, rest, satisfaction, defense, and other associations of green are appropriate at the end of the process cycle, when the outcome of the Action is assessed and reflected upon.
- Blue: Novelty
  Discretion, intelligence, clarity, cooling, focusing, and concentrating qualities specific to blue facilitate recognition of Novelty at the beginning of the process cycle.

The color code of the primary process stages thus consists of the sequence of blue followed by red followed by green. Intermediate stages Goal-plan, Progress, and Perception, then correspond to magenta (purple, violet), yellow, and cyan. This results in the standard chromatic sequence cyan—blue—magenta—red—yellow—green (McCamy, 1993), distinctly seen in Figure 4.

The colors of this list symmetrically align with Osgood's semantic factors (Section 3.3). Red (fire, blood) is the most active and warm, as opposed to cyan (ice, sky) which is the most passive and cold. Bluish-magenta is the most tense and potent, while yellowish-green is the most relaxed and peaceful. The *temperature* and *tension* axes of the chromatic plane then align with the *activity* and *potency* axes of the qubit's azimuthal plane shown in Figure 2B.

### 4.2. Encoding of main colors

**Figure 5:**
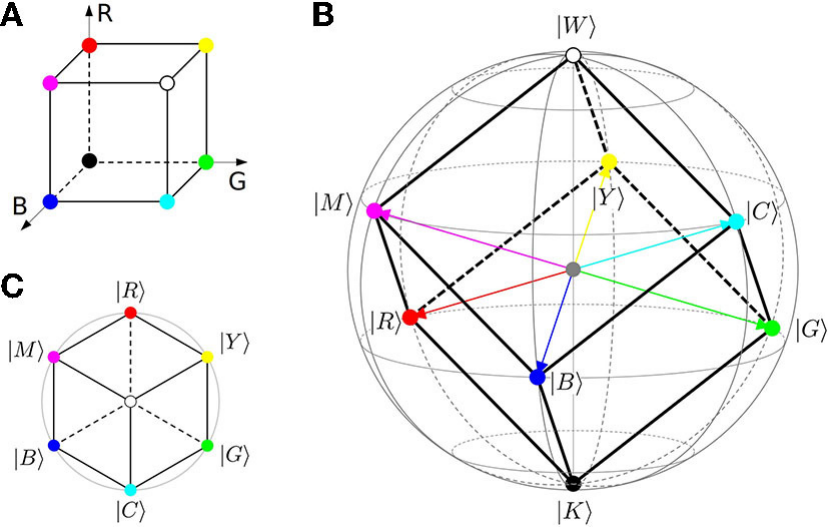
Building of the qubit-color space. RGB color cube **(A)** is inscribed in the Bloch sphere **(B)** such that white (W) and black (K) map to the basis states |1〉 and |0〉. Primary RGB (Equation 11) and secondary CMY (Equation 13) colors define horizontal planes, dividing the diameter of the sphere into three equal parts. **(C)**: View from the black-white diagonal. Main chromatic colors form the rainbow sequence seen in the equator of the HSL bi-cone (Figure 4).

Mapping of main colors to the qubit states relies on the RGB cube model, representing any color as a mixture of red, green, and blue with weights ranging from 0 to 1. As shown in Figure 5, the cube (A) is inscribed in the Bloch sphere (B) such that white (W) and black (K) take place of the north and south poles, respectively.

Qubit states of the RGB colors are found from their positions in the Bloch sphere, having the same polar angle θ = arccos(1/3) ≈ 1.23 ≈ 70.5° as indicated in panel (B) by latitude line. Azimuthal phases of these colors are defined by their mapping to the primary process stages described in Section 4.1, namely $\phi_{\text{blue}}=60{}^{\circ}=\pi /3$, $\phi_{\text{red}}=180{}^{\circ}=\pi$, $\phi_{\text{green}}=300{}^{\circ}=5\pi /3$. The resulting qubit states are


(11) $$\begin{array}{l} |R\rangle =\frac{1}{\sqrt{3}}\left[\begin{array}{c} \sqrt{2}e^{i\pi }\\ 1 \end{array}\right]\\ |G\rangle =\frac{1}{\sqrt{3}}\left[\begin{array}{c} \sqrt{2}e^{5i\pi /3}\\ 1 \end{array}\right]\\ |B\rangle =\frac{1}{\sqrt{3}}\left[\begin{array}{c} \sqrt{2}e^{i\pi /3}\\ 1 \end{array}\right]. \end{array}$$

These states^3^ are not orthogonal, with each one expressed through the rest two as


(12) $$\begin{array}{l} e^{-i\pi /3}|B\rangle +e^{i\pi /3}|R\rangle =|G\rangle \\ e^{-i\pi /3}|R\rangle +e^{i\pi /3}|G\rangle =|B\rangle \\ e^{-i\pi /3}|G\rangle +e^{i\pi /3}|B\rangle =|R\rangle . \end{array}$$

Symmetric real-valued superpositions of states (Equation 11)


(13) $$\begin{array}{l} \frac{|R\rangle +|G\rangle }{\sqrt{2}}=\frac{1}{\sqrt{3}}\left[\begin{array}{c} e^{4i\pi /3}\\ \sqrt{2} \end{array}\right]=|Y\rangle \\ \frac{|R\rangle +|B\rangle }{\sqrt{2}}=\frac{1}{\sqrt{3}}\left[\begin{array}{c} e^{2i\pi /3}\\ \sqrt{2} \end{array}\right]=|M\rangle \\ \frac{|G\rangle +|B\rangle }{\sqrt{2}}=\frac{1}{\sqrt{3}}\left[\begin{array}{c} 1\\ \sqrt{2} \end{array}\right]=|C\rangle \end{array}$$

are yellow (Y), magenta (M), and cyan (C), occupying another horizontal level θ = arccos(−1/3) ≈ 1.91 ≈ 109.5° with phases $\phi_{\text{cyan}}=0=0{}^{\circ}$, $\phi_{\text{magenta}}=2\pi /3=120{}^{\circ}$, $\phi_{\text{yellow}}=4\pi /3=240{}^{\circ}$. The corresponding location of states (Equation 13) on the Bloch sphere is exactly as prescribed by the cube geometry, shown in Figure 5B.

As expected for complementary colors, cyan is the opposite of red, yellow is the opposite of blue, and magenta is the opposite of green. The qubit encoding expresses this by orthogonality relations


(14) $$\begin{array}{l} \langle R|C\rangle =\langle G|M\rangle =\langle B|Y\rangle =0, \end{array}$$

following from Equations (11), (13), and definition (Equation 4). In terms of the corresponding Stokes vectors (Equation 8), orthogonalities (Equations 14, 15) become oppositions, so that cyan is precisely “minus red” ${\vec{S}}_{\text{cyan}}=-{\vec{S}}_{\text{red}}$, magenta is “minus green,” yellow is “minus blue,” and black is “minus white” (McCamy, 1993).

White, finally, is the symmetric composition of either the main or complementary color triad:


(15) $$\begin{array}{l} |W\rangle =\left[\begin{array}{c} 0\\ 1 \end{array}\right]=\frac{|R\rangle +|G\rangle +|B\rangle }{\sqrt{3}}=\frac{|C\rangle +|M\rangle +|Y\rangle }{\sqrt{6}},\\ \langle W|K\rangle =\langle 1|0\rangle =0. \end{array}$$

Equivalently, the whole derivation could start from the complementary triad (Equation 13), producing R, G, B, and K as derivatives.

### 4.3. Full map

Besides eight vertices of the color cube considered above, the only colors with obvious qubit-state representation (of classical type Equation 9) are shades of gray, mapped to the Z-axis of the Bloch sphere. The color of an arbitrary qubit state derives from *real-valued* decomposition of a pure state (Equation 1) in the RGB basis (Equation 11)


(16) $$|\psi \rangle =\tilde{r}|R\rangle +\tilde{g}|G\rangle +\tilde{b}|B\rangle ,\;\tilde{r},\tilde{g},\tilde{b}\in \mathbb{R}$$

as explained in the Appendix. In the resulting map, any mixed state $\hat{\rho }$ (Equation 7) has RGB color with components 0 ≤ *r, g, b* ≤ 1


(17) $$\begin{array}{l} \left\{r,g,b\right\}=\frac{1}{2}\left(1+|\vec{S}|\frac{\left\{\tilde{r},\tilde{g},\tilde{b}\right\}}{\text{max}\left(\left|\tilde{r}|,|\tilde{g}|,|\tilde{b}\right|\right)}\right), \end{array}$$

where $\vec{S}$ is the Stokes vector, representing state $\hat{\rho }$ (Equation 8) with coordinates *x*, *y*, *z*, and


(18) $$\begin{array}{l} \begin{array}{c} \tilde{r}=\frac{z+2\sqrt{2}y}{\sqrt{3}},\\ \tilde{g}=\frac{z-\sqrt{2}y-\sqrt{6}x}{\sqrt{3}},\\ \tilde{b}=\frac{z-\sqrt{2}y+\sqrt{6}x}{\sqrt{3}} \end{array} \end{array}$$

are real-valued *spherical* RGB components such that


(19) $$\begin{array}{l} {\tilde{r}}^{2}+{\tilde{g}}^{2}+{\tilde{b}}^{2}\leq 1. \end{array}$$

Point $\tilde{r}=\tilde{g}=\tilde{b}=0$, nullifying denominator in Equation (17), corresponds to the center of the Bloch sphere *x* = *y* = *z* = 0. The map is one-to-one and full, representing unique colors by unique qubit states and fully covering both the RGB color cube and the qubit state space.

**Figure 6:**
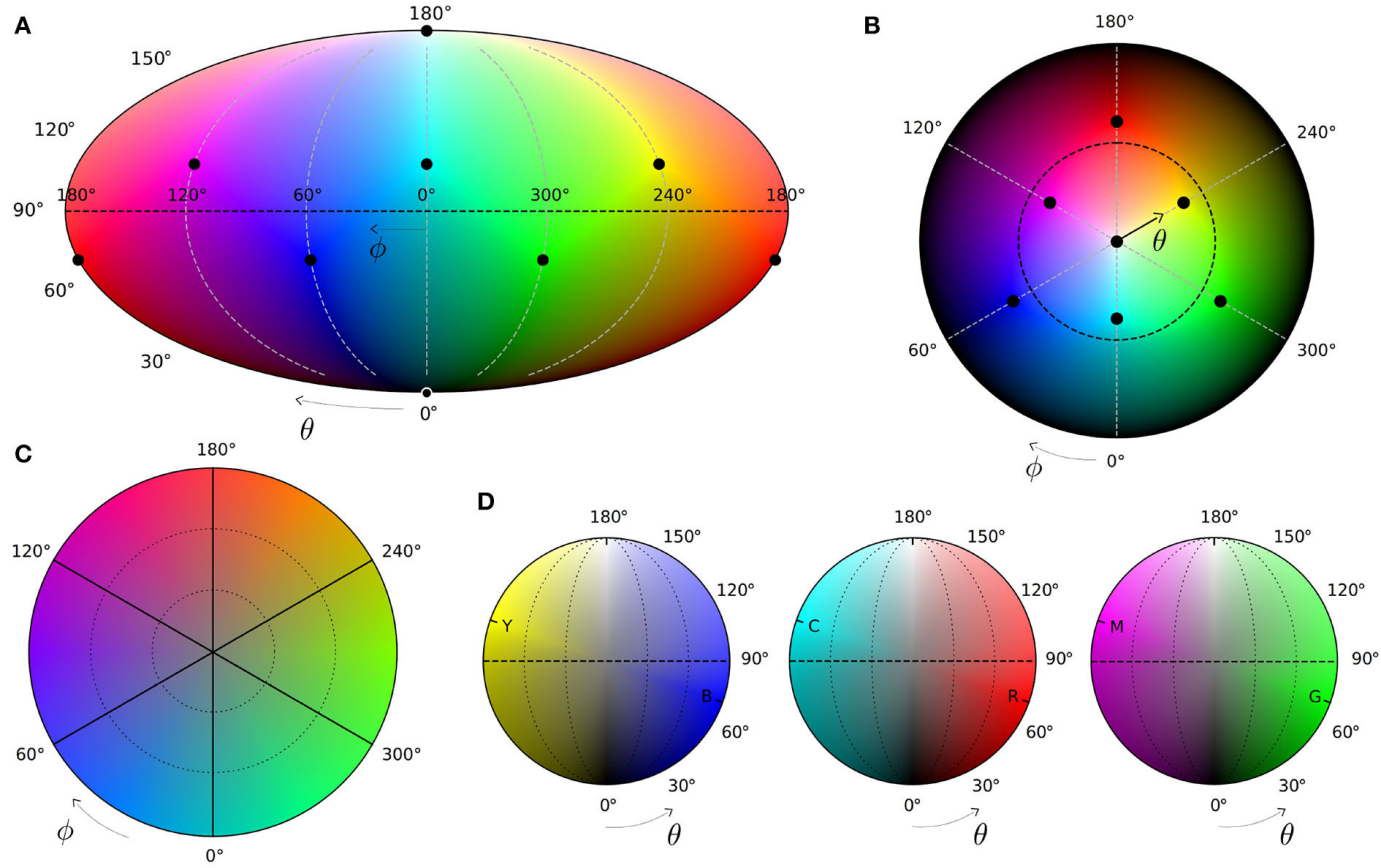
Color map of the qubit state space (Equation 17). Top: pure colors on the surface of the Bloch sphere in Mollweide **(A)** and polar **(B)** projections. Dots locate eight main colors (Equation 11), 13, and 15) in the vertices of the color cube (Figure 5), dashed lines show the equatorial plane. Bottom: mixed colors inside the Bloch sphere in equatorial section **(C)** and three vertical sections **(D)** going through solid lines in panel **(C)** and opposite pairs of the main chromatic colors. Dotted lines show elliptical surfaces of constant saturation 1/3 and 2/3. Produced by Matplotlib 3.3.4 (Hunter, 2007).

Map (Equation 17) is illustrated in Figure 6. Panel A is Mollweide projection of the sphere's surface, with dots showing the location of eight main colors. Panel (B) shows the same surface in spherical coordinates, with the radius being polar angle θ. Accordingly, the North pole (white) locates at the center of the circle, while the South pole (black) maps the perimeter. In both (A) and (B) the equator θ = π/2 is shown by the dashed line.

The bottom part of Figure 6 shows mixed colors inside the Bloch sphere in planar sections. Panel C shows the equatorial section, in which diameter (Z-axis) corresponds to the central point of gray color *r* = *g* = *b* = 0.5. Panel D shows vertical sections along the solid lines of the Panel C. Each section includes two main chromatic colors (red and cyan for ϕ = 0, green and magenta for ϕ = 120°, blue and yellow for ϕ = 240°). In each case, dashed lines denote the equatorial plane, and vertical is the Z-axis of the Bloch sphere, accommodating shades of gray from black to white.

### 4.4. Properties

The rest of this section discusses features of the developed qubit-color map, including symmetries and prototype structure.

#### 4.4.1. Mathematical viewpoint on basic colors

The qubit model provides insight into the possible decomposition of colors. Unlike standard approaches noted in Section 3.2, the number of basic colors appears to be fixed by the mathematics in use.

##### Two

Standard quantum-theoretic form (Equation 1) exemplifies the decomposition of qubit-color states with complex-valued coefficients. In this case, any pure color is represented as a coherent superposition of black |0〉 and white |1〉, or any other pair of orthogonal states like (Equation 14). The first line of Equation (11), e.g., means that red is produced by superposing $\sqrt{1/3}$ of white and $-\sqrt{2/3}$ of black^4^. Other colors are partially-coherent mixtures of the same basis as described in Section 2.4.

##### Three

Limiting coefficients to real numbers requires one more basis color, as exemplified by decomposition (Equation 16) of arbitrary qubit-color state in |*R*〉, |*G*〉, and |*B*〉 components. This decomposition aligns with the physiological mechanics of color vision. As in standard RGB logic, real-valued decomposition weights $\tilde{r},\tilde{g},\tilde{b}$ could be encoded by positive intensities (frequency rates of neural firing) of the corresponding chromatic receptors in the retina^5^.

Six possible orderings of decomposition amplitudes (Equation 17) (*b* > *g* > *r*, *b* > *r* > *g*, etc.) divide azimuthal range 0 ≤ ϕ < 360° to six equal sectors of 60° each. This is the color counterpart of process stages (Section 2.2), centered at junctions between these sectors. In this sequence, main RGB colors map to three maxima in the third harmonics of affective value, while intermediate CMY colors correspond to the minima (Guilford, 1934).

Three basic hues vividly illustrate the triadic nature of qubit-type semantics (Surov, 2021). In the qubit encoding, semantic relations between archetypal contexts of red, green, and blue classes take the circular form (Equation 12) typical for semantic triads (ibid.). Measuring color relations by the triad-ratio method (Helm, 1964; Komarova and Jameson, 2013; Liu and Heer, 2018) accounts for this fundamental feature.

##### Four and more

A number system that could decompose qubit in more than three basis states does not come to mind. Without such mathematics, four, five, and more basic hues, used in some models (Rossi and Buratti, 2015), although physiologically possible (Jacobs, 2018), are conceptually redundant. This, of course, does not limit the taxonomy of color terms, defining granularity of the qubit-semantic space^6^.

#### 4.4.2. Spatial properties

##### Color space in the real sense

Any normalized superposition or mixture of qubit-color states also is a valid qubit-color state. In contrast to HSL and other arbitrarily constructed models of color, linear algebra of Hilbert space qualifies this representation of color as space in the true mathematical sense. Far from the quantum-theoretic argument, the spherical geometry of this representation was envisioned by Wundt (1897, ch. I:19), A. S. Forceus Shamey and Kuehni (2020, ch. 17), P. O. Runge, M.-E. Chevreul, and others (Kay and McDaniel, 1978; Rossi and Buratti, 2015).

Neither spherical topology, nor triadicity of color semantics conflict with the definition of color by pairs of orthogonal dimensions, as suggested e.g. by the opponent color theory (Section 3.2). As shown by linear axes in Figure 4, this approach discloses dimensions of *tension* and *warmth*, aligned with classical EPA space (Section 3.3).

While confirming opposition of yellow and blue, the present model, however, suggests that red and green are opposite not to each other, but to cyan and magenta (Equation 14), as previously noted by McCamy (1993), Jameson and D'Andrade (1997), and Conway (2009). Additionally, all three chromatic oppositions are not orthogonal to the black-white axis as seen from Figures 5B, 6D. Exactly orthogonal dimensions are, e.g., XYZ axes of the Bloch sphere shown in Figure 1.

The qubit color space supports metrics of standard quantum theory (Nielsen and Chuang, 2010; ch. 9.2). *Trace distance* between two arbitrary mixed states, in particular, is equivalent to Euclidean distance between the corresponding Stokes' vectors (Equation 8). This establishes correspondence of the qubit representation with classical studies (Helm, 1964; Indow, 1980, 1988; Indow and Aoki, 1983), mapping colors to three-dimensional Euclidean space by multidimensional scaling of color-difference judgments.

##### Symmetries

By keeping mutual positions of eight basic colors in vertices of the RGB color cube as shown in Figure 5, the obtained model maintains several conceptual symmetries.

Eight corners of the cube form two regular tetrahedrons, including RGBW and CMYK vertices, respectively. The former originates directly from the types of light-sensitive cells in the retina. Namely, red, green, and blue stand for three types of cone cells^5^, while white corresponds to the rod cells with sensitivity peaked at 498 nm, the signal of which is experienced as achromatic lightness. Emotionally, vertices of this tetrahedron stand for the minimal set of four main classes: white for all positive, red for anger, green for sadness, and blue for fear (Jack et al., 2016).

The remaining vertices with orthogonal qubit representations (Equations 14, 15) form a complementary tetrahedron of subtractive CMYK colors, opposite to the primary (additive) one and pointing down in Figure 5. Both tetrahedrons achieve uniform coverage of the qubit color space by four prototypes, facilitating their technological and physiological use (McCamy, 1993; Fortner and Meyer, 1997; Regier et al., 2007).

In projection to the chromatic (horizontal) plane, two tetrahedrons form a regular hexagon shown in Figure 5C, representing the standard rainbow sequence without orange. The symmetry of this system, crucial for practical use (McCamy, 1993; Fortner and Meyer, 1997; Regier et al., 2007), requires exactly three primaries and three complements; dropping any of them breaks this structure, fundamental to the qubit semantics. Omission of cyan (Russian “goluboy” Paramei, 2005; Winawer et al., 2007), for example, leads to the five-based Munsell system (Section 3.2), missing the triadic symmetry. The additional dropping of magenta then results in the asymmetric color space based on the RGBY four (Palmer, 1999).

##### Disentanglement of lightness from hue

Retaining cubical positions of basic colors also solves the orthogonality problem of the HSL model, noted in Section 3.4. In contrast to the standard HSL, qubit representation maps RGB and CMY colors to both sides of the equatorial plane as shown in Figure 6, respecting their difference in lightness. In agreement with the standard coding, RGB (100, 010, 001) and CMY (011, 101, 110) colors have one-third and two-thirds of maximal lightness, dividing the diameter of the Bloch sphere into three equal parts. The resulting difference is seen by comparing the lightness of colors in Figure 6C with the equatorial circumference of the HSL color solid, shown in Figure 4.

The achieved disentanglement of perceptual lightness from hue facilitates the development of more efficient image-processing algorithms and graphical interfaces (Burns and Shepp, 1988; Chen et al., 2007). Together with a proper account for color semantics, this allows, e.g., the construction of better color maps for various domains of data analysis (Borland and Taylor Ii, 2007; Wang et al., 2008; Larrea et al., 2010; Zhou and Hansen, 2016; Bujack et al., 2018; Schloss et al., 2019; Reda and Szafir, 2021; Zhang et al., 2021). When restricted to a single dimension, however, the standard color circle is arguably the most illustrative scheme of pure chromatic colors (McCamy, 1993).

##### Relation to QHSL

The above theory is related to the recently proposed quantum-HSL model (Yan et al., 2015, 2021c), representing color by qubit states as mentioned in Section 3.3. This approach also maps the hue to the circular phase dimension ϕ of the qubit, sectioned into six main chromatic colors in the symmetric configuration shown in Figure 5C. In contrast to the present model, however, polar angle θ quantifies saturation of the color, while lightness is encoded in an additional sequence of qubits. This and similar representations allow the design of advantageous algorithms for the storage, processing, and retrieval of graphic information (Yan et al., 2016, 2017).

In comparison with this approach, the present model encodes all dimensions of color in a single qubit by using mixed states within the Bloch sphere in addition to its surface. While also compatible with the standard quantum-technological toolbox, this format is a more compact representation, properly reflecting semantics of color. Together with disentangling of chromaticity from perceptual lightness mentioned above, this facilitates further advances in quantum-inspired methods of data analysis.

## 5. Unified space of emotion and color

As seen from Table 1, semantics of color is largely described in terms of subjective states, classified as emotions and emotion-related states of subjective experience (Fehr and Russell, 1984). On the other hand, emotions themselves derive from qubit semantics as shown in Section 2. Through the color map developed above, Section 5.1 establishes a three-side correspondence between color, emotion, and qubit states. Section 5.2 discusses the resulting possibilities for cross-disciplinary interaction.

### 5.1. Mapping colors to emotions

The correspondence between colors and emotions is defined by the process-stage allocation of both, made in Sections 2.3 and 4.1. Namely, **blue** associates with emotions accompanying the analysis of Novelty, **red** maps the experiences of Action, while **green** corresponds to the emotional estimation of the Result. Full correspondence, linking each of the six chromatic colors to positive and negative experiences, is summarized in Table 2.

**Table 2:** Correspondence of colors, process-semantic classes, and emotional experiences.

**Color**	**Process stage**	**Positive emotions**	**Negative emotions**
Cyan	Perception	Calmness, serenity, bliss	Depression, shame, guilt
Blue	Novelty	Surprise, interest	Anxiety, startle, fear
Magenta	Goal-plan	Inspiration, ambition	Irritation, stress
Red	Action	Passion, bravery, zeal	Anger, fury, rage
Yellow	Progress	Acceptance, delight	Disappointment, disgust
Green	Result	Joy, contentment, rapture	Sadness, grief, despair

#### Color-emotion sphere

**Figure 7:**
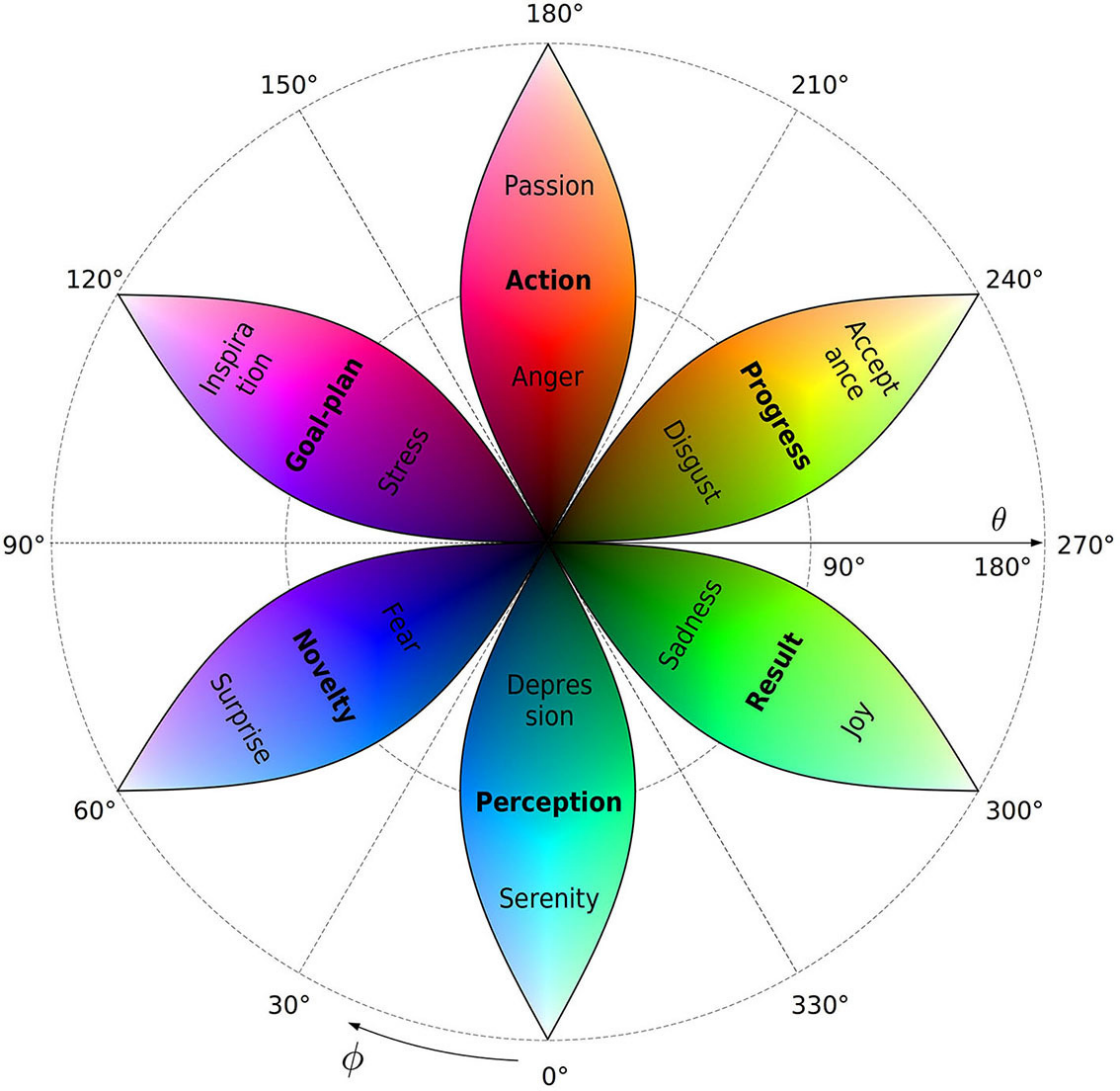
Color-emotion map of the qubit semantic space in the planar layout of the Bloch sphere. The leaves are centered at the main chromatic colors used above. Each leaf is the corresponding area-preserving part of the color map shown in Figure 6. The polar angle increases outwards, such that the South pole |0〉 is mapped to the center θ = 0, while the North pole |1〉 locates at the outer circle θ = 180°. Process stages (bold), the corresponding positive and negative emotional prototypes locate in the middles, inner (θ < 90°), and outer (θ > 90°) parts of the leaves according to Figure 3 and Table 2.

Table 2 is visualized by overlapping the qubit-color map, Figure 6, with the qubit-emotion sphere, Figure 3. The resulting map is shown in Figure 7. This is a planar layout of the Bloch sphere, with the azimuthal dimension ϕ categorized into six process-semantic sectors as before. In this view, each sector becomes a leaf-shaped area of width π/3*sinθ, defined by spherical geometry.

In terms of color, this is an area-preserving version of Figure 6B with reversed radial dimension. Accordingly, the south pole |0〉—black color and negative limit of all emotional classes—locates in the center, while the north pole |1〉—white color and positive limit of emotions—maps to the periphery. Process-stage labels Perception, Novelty, Goal-plan, Action, Progress, and Result are placed in their respective leafs near the equatorial circle θ = 90° due to their evaluative neutrality.

Parallel demarcation of the qubit state in categories of emotion and color, both being genuinely subjective, semantic-class phenomena (Alcaro et al., 2017; Yanshin, 2018), suggests asking which one is more fundamental. In contrast to emotion, typically associated with psychology of complex organisms, color has an objectively-measured physical primitive—a wavelength of light. This nominates color as the primary semantic code of Nature (Yanshin, 1996), subsequently hardwired into the physiology of vision and neural architectures of various forms of life (Sokolov, 2001a,b). Monochromatic, dichromatic, tetrachromatic, and more complex vision systems then may be seen as Nature's attempts to develop and use alternative structures of meaning.

#### Relation to Plutchik's model

The obtained correspondence between color and emotional classes has a superficial similarity with popular wheel-type schemes, ascribing basic emotions to specific positions in the color circle (Karimova, 2021). Plutchik's emotion solid, in particular, is close to the present model in mapping emotional states to a three-dimensional half-sphere structure with azimuthal sectors colored in natural order (Plutchik, 1958, 2001). Although superficially similar, this mapping is conceptually different from the present article.

The main contrast is the location of basic emotions in the azimuthal dimension. With shaky logic behind it, Plutchik's ordering (disgust—expectancy—anger—joy— acceptance—surprise—fear—sadness) has little agreement with the experiment (Plutchik, 1980). The present approach explains this by disparate nature of four basic opposites, underlying this order (Kellerman, 2020; ch. 5). Namely, *joy-sadness* and *acceptance-disgust* pairs are opposite in valence within the same process-stage classes, while *anger-fear* and *expectancy-surprise* differ in process stage rather than in valence. Accordingly, qubit representation distinguishes these states in different spherical coordinates instead of a single circular dimension.

In agreement with the semantics of colors (Section 3.1) this allows, in particular, to map *fear* to (dark) blue and *sadness* to (dark) green, in contrast to the reverse order postulated in Plutchik (2001). The scheme shown in Figure 7 is thus more coherent factually, conceptually, and mathematically. Plutchik's model, however, takes credit as an inspiring conjecture used in several research directions (Wang and Pereira, 2016; Gu et al., 2019; Yan et al., 2021b).

#### Relation to Sokolov's 4D-sphere

The developed model relates to the space of color-emotion categories proposed by Kozlovskiy et al. (2016) and Kiselnikov et al. (2019), also establishing correspondence between the two domains. Unlike the qubit model, however, this approach maps hue, saturation, and lightness to three angular dimensions, locating emotions and colors on the surface of a four-dimensional sphere (Izmailov and Sokolov, 1992; Sokolov and Boucsein, 2000; Leonov and Sokolov, 2008).

Although different geometrically and semantically, this representation is close to the qubit model in a crucial aspect of conceptual significance. Sokolov's color-emotion sphere is considered as a special instance of a fundamental cognitive architecture, encoding neuronal states by vectors of constant length. The neural basis of trichromatic vision just exemplifies this universal coding, central to other functions of human intelligence (Izmailov et al., 1989; Sokolov, 2000, 2001a). The present approach is of similar generality, at the same time introducing conceptually new aspects, unique to quantum semantics (Surov, 2022).

### 5.2. Connecting emotion science and quantum cognition

In previous decades, emotion science and quantum models of cognition and decision were developing independently (Khrennikov, 2010; Busemeyer and Bruza, 2012; Fontaine et al., 2013; Barrett et al., 2016). Advantage of quantum theory in describing irrational decisions and the central role of affect in human cognition (Duncan and Barrett, 2007; Dukes et al., 2021), however, would suggest the opposite. This section indicates the points of possible interaction, allowed by the color link developed above.

#### Quantum methods for emotion science

Psychological studies of emotion and color lack conceptually-quantitative expression, connecting them to observable phenomena. The resulting encapsulation in the cognitive realm deprives the models of practical application, undermining their fundamental value. The obtained result addresses this problem by situating emotion and color in the broader scope of behavioral semantics.

Color, in particular, appears as the natural encoding of qubit-semantic states, affecting objectively measurable regularities of decision making, quantified by the quantum probability theory. This setting resonates with the goals pursued in robotics and artificial intelligence (Breazeal, 2003; Cavallo et al., 2018; Pessoa, 2019b; Samsonovich, 2020b; Deng et al., 2021; Kotov et al., 2021; Yan et al., 2021a,b; Samsonovich, 2020a; ch. 7, 10, 13, 34, 54, 56, 57): as in natural cognitive systems, subjective states of individuals, essentially, couple stimuli to responses in a personally meaningful way, as required for non-trivial behavior. The established equivalence of color and emotion encodings of subjective meaning strengthens this practical approach. Emotion coloring (Izard, 1977; Pinker, 2008; Russell, 2009; Khrennikov, 2021), social color, color of information excitations (Khrennikov, 2018; Khrennikov et al., 2018), and money (Ferraris, 2019; Orrell, 2021), in particular, turn from insightful metaphors to rigorous mathematical correspondence, throwing light on empirically-discovered phenomena of practical interest (Bellizzi and Hite, 1992; Kwallek et al., 1996; Aslam, 2005; Hill and Barton, 2005; Doyle and Bottomley, 2006; Elliot et al., 2007; Labrecque et al., 2013; Neville, 2022).

Productive steps in this direction are, in fact, already made in quantum models of cognition and decision, developed in recent decades. In many cases, the correspondence is established simply by changing terminology from “cognitive” to “affective” states. Applied emotion science is then recognized in the existing models of cognitive fallacies and irrational decision-making (Busemeyer et al., 2011; Pothos and Busemeyer, 2013; Ashtiani and Azgomi, 2015), subjective utility (Basieva et al., 2018), non-classical social and economic behavior (Khrennikov, 2016; Njegovanovic, 2018; Meghdadi et al., 2022), semantics of natural language and information retrieval (Aerts et al., 2013; Melucci, 2015; Surov et al., 2021), conceptual and belief networks (Gabora et al., 2008; Moreira et al., 2020), cybernetics, artificial intelligence, and knowledge representation (Wolff et al., 2018; Bickley et al., 2021; Deng et al., 2021; Yan et al., 2021a). Color-emotion research, thus, gets a natural connection to vast areas of applied science and technology, at the same time acquiring the requested conceptual and theoretical basis (Brower, 1949; de Gelder, 2017; Reisenzein, 2019; Burghardt and Bodansky, 2021; Mascolo, 2021; Uher, 2021).

#### Emotion science for quantum cognition

As inherited from physics (Svozil, 2018), a distinctive feature of quantum cognition is the lack of commonly accepted interpretation. The approach largely develops in a mathematically-formal way, detached from physical and psychological perspectives—a sort of theoretical black box, producing observable probabilities for particular behavioral cases without explaining them (Blutner and beim Graben, 2016; Sozzo, 2019). Although methodologically safe, this stance hinders the progress of the field, where productive models are often found by blind search and typically lack the predictive power, needed for practical use.

The obtained result suggests an approach to this problem. Color interface, for example, allows using standard methods of psychological diagnostics to find qubit's phase parameters in quantum models of cognition and decision, necessary to use them in predictive mode (Surov et al., 2019; Surov, 2021; Shan, 2022). Besides application to qubit-state models, the established color-emotion structure can facilitate interpretation of more complex wavefunctions, observables, projection and transformation operators, and other mathematical instruments, often having no clear psychological counterpart. This would open access to other results and methods of cognitive science and psychology, continuing a long history of productive interaction (Khrennikov, 2015).

## 6. Outlook

###  Semantic view of emotion and color

Three-sided isomorphism of qubit semantics with color and emotional states bears fundamental implications. The three appear not as separate phenomena or models, but as alternative encodings of a single underlying phenomenon—an elementary subjective experience. This phenomenon, called *core affect* (Russell and Barrett, 1999), is the essence of the subjectively-semantic, “psychological” dimension of Nature, noted in the Introduction (Surov, 2022).

This broader perspective allows one to give psychological definitions of emotion and color, filling a problematic lacuna (Yanshin, 1999; Reisenzein, 2007; Dixon, 2012; Pessoa, 2019a). Color, in particular, can be defined as visual encoding of elementary subjective experience, while emotion encodes this experience through innate psycho-physiological scripts (Demos, 1995; Panksepp et al., 2017). Qubit states, on the other hand, encode the same thing in abstract mathematical form, allowing for quantitative behavioral modeling.

###  The qubit as a semantic atom

Aforementioned elementarity of subjective experience, refers to its derivation from the simplest possible, binary uncertainty, faced by an individual. Only in this case, the corresponding quantum state aligns with the empirically discovered structures of emotion and color, which are considered as psychological primitives (Yanshin, 1996; Barrett and Bliss-Moreau, 2009; Alcaro et al., 2017). In analogy with basic blocks of matter, the qubit then appears as a model of the *semantic atom*, representing an elementary unit of affective meaning in Nature.

The concept of semantic atom aligns with several existing approaches. Long before the separation of physics from spirit, famous Greeks considered atoms as elements of both body and soul (Bailey, 1928). In the present version, however, semantic atom is an exclusively informational structure (which, of course, does not imply its existence without material carrier). This is, simultaneously, the quantum of affective distinction (Volchenkov, 2010) and the template for a personal Umwelt, through which a subject experiences one's individual becoming (Surov, 2022). Stressing its qualitative nature (i.e., the impossibility of direct measurement), analogous thing called *quale* (Palmer, 1999; Haikonen, 2009; Beshkar, 2022) is a unit of recently considered “qualia space” (Balduzzi and Tononi, 2009; Resende, 2022), ascending to Jamesian “mind-stuff” (James, 1890; ch.VI) and Riemannian “mind mass” (Riemann, 1900).

The developed model thereby opens a fresh perspective on psychic atomism both in its original and modern (Eccles, 1990; Khrennikov et al., 2018; Bell et al., 2021) versions. Parallel to the diversity of material atoms there could be other types of semantic ones, represented by various wavefunctions of quantum theory. The complexity of human psychology then could be due to this variety of atoms and possible semantic bonds, forming our affective medium parallel to the chemical composition of matter. Semantic chemistry of this medium is an uncharted terrain, open for investigation.

## Data availability statement

The original contributions presented in the study are included in the article/supplementary material, further inquiries can be directed to the corresponding author.

## Author contributions

IS prepared the manuscript and contributed to the article and approved the submitted version.

## Funding

This research was funded by grant of Russian Science Foundation (Project Number 20-71-00136).

## Conflict of interest

The author declares that the research was conducted in the absence of any commercial or financial relationships that could be construed as a potential conflict of interest.

## Publisher's note

All claims expressed in this article are solely those of the authors and do not necessarily represent those of their affiliated organizations, or those of the publisher, the editors and the reviewers. Any product that may be evaluated in this article, or claim that may be made by its manufacturer, is not guaranteed or endorsed by the publisher.

## Footnotes

^1^Fundamentally, this perspective stems from the recognition of two fundamentally different kinds of the *lack of knowledge* (Aerts, 1999), or, equivalently, refinement of the concept of *uncertainty* to classical and quantum types (Surov, 2021). Classical type includes uncertainties about actual states of Nature and states, predetermined to happen in the future. Such is, e.g., uncertainty about the exact amount of money in one's pocket and uncertainty about a train's arrival time for someone ignorant of its schedule. Quantum uncertainty, in contrast, is not due to a subjective lack of knowledge. This is the objective *potentiality* that can be actualized only by experiment, bringing the individual to a new state in which the observable quantity takes definite value. The capability to resolve quantum uncertainties is the essence of decision-making practice that differs living behavior from inert mechanics.

^2^A differential mechanism behind this model is found at a particular stage of neural processing of visual data. It provides, for example, a simple explanation of afterimages (after prolonged fixation on any of RGBY colors, white is temporally perceived as its pair), less expected from three-based models (cf. Jameson and D'Andrade, 1997; Wooten and Miller, 1997).

^3^Unlike states in Equations (1) and (2), phase factors *e*^*iϕ*^ are ascribed to the |0〉 component of the vectors for mathematical convenience. This conversion, also used in the Appendix, is theoretically safe.

^4^Agreeing with Goethe's insight about the active role of darkness in subjective experience of color, as demonstrated e.g., in perceptual experiments with shadow (Goethe, 1840, p. 69, 239:240). In the quantum approach, colors indeed can be seen as (complex-valued) degrees of darkness and light.

^5^Normal physiology of human vision involves three chromatic receptors being cone-shaped cells in the retina, with sensitivities centered at 564 nm for red, 534 nm for green, and 420 nm for blue types (Bowmaker and Dartnall, 1980; Shevell, 2003; Conway, 2009). Activation of these receptors, processed by differential schemes (Dedrick, 1998, chap. 2), underlines our perception of hue.

^6^After white and black, present in all known taxonomies, chromatic colors are introduced to more and more developed languages in the following order: red, green, yellow, blue, brown, and then set of purple, pink, orange, and gray (Berlin and Kay, 1975; Kay and McDaniel, 1978).

## Appendix: Derivation of the qubit-color map

Mapping (Equations 17, 18) is based on a real-valued decomposition of arbitrary pure qubit (Equation 1) (multiplied by phase factor *e*^−*iϕ*^ for algebraic convenience) into red, green, and blue vector-states (Equation 11)

(20)
$$|\psi_{1}\rangle =\left[\begin{array}{c} e^{-i\phi }\cos\frac{\theta }{2}\\ \sin\frac{\theta }{2} \end{array}\right]=\tilde{r}|R\rangle +\tilde{g}|G\rangle +\tilde{b}|B\rangle ,\;\tilde{r},\tilde{g},\tilde{b}\in \mathbb{R},$$

so that

(21a)
$$\begin{array}{l} \sin\frac{\theta }{2}=\frac{\tilde{r}+\tilde{g}+\tilde{b}}{\sqrt{3}}, \end{array}$$

(21b)
$$\begin{array}{l} e^{-i\phi }\cos\frac{\theta }{2}=\sqrt{\frac{2}{3}}\left(\tilde{r}e^{i\pi }+\tilde{g}e^{-i\pi /3}+\tilde{b}e^{i\pi /3}\right). \end{array}$$

Normalization of the right-hand side of Equation (20) restricts coefficients $\tilde{r}$, $\tilde{g}$, $\tilde{b}$ to the surface of a unit sphere (Equation 19). Section of this sphere by plane (Equation 2a) produces a circle, in which the solution for Equation (20) is a point, defined by phase ϕ. For θ = 0, this circle is equatorial circumference, while limiting cases θ = ±π correspond to polar points $\tilde{r}=\tilde{g}=\tilde{b}=\pm 1/\sqrt{3}$.

The original qubit (Equation 1), however, is limited to nonnegative range 0 ≤ θ ≤ π, covering only half of the sphere (Equation 19). Preservation of symmetry and inclusion of mixed states is achieved by means of a second qubit

(22)
$$\begin{array}{l} {\hat{\rho }}_{2}=\frac{1}{2}\left[\begin{array}{cc} 1-\sin\frac{\theta }{2} & \cos\frac{\theta }{2}e^{-i\phi }\\ \cos\frac{\theta }{2}e^{i\phi } & 1+\sin\frac{\theta }{2} \end{array}\right] \end{array}$$

with elements of the matrix taken from Equations (21). Identification of the resulting state (Equation 22) with the latter form of Equation (7) produces the corresponding spherical color components (Equation 18). Standard RGB weights are obtained by transformation (Equation 17), converting sphere (Equation 19) to a unit cube and shifting it to the positive quadrant.
